# Two new species and one subspecies of *Craniophora* Snellen, 1867 (Lepidoptera, Noctuidae, Acronictinae) from China

**DOI:** 10.3897/zookeys.353.5990

**Published:** 2013-11-20

**Authors:** Ádám Kiss, Péter Gyulai

**Affiliations:** 1 University of Debrecen, Department of Evolutionary Zoology and Human Biology, Egyetem tér 1, H-4032 Debrecen, Hungary; 2 H-3530 Miskolc, Mélyvölgy 13/A, Hungary

**Keywords:** New taxa, Noctuidae, Acronictinae, *Craniophora*, China, Fujian, Hainan, Sichuan

## Abstract

Three *Craniophora* taxa from China, *C. fujianensis* Kiss and Gyulai, **sp. n.**, *C. fujianensis hainanensis* Kiss and Gyulai, **ssp. n.** and *C. sichuanensis* Kiss, Gyulai and Saldaitis, **sp. n.**, are newly described. Adult habitus and male genitalia are illustrated and compared with those of *C. harmandi* (Poujade) and *C. praeclara* (Graeser). Females of the new taxa are unknown.

## Introduction

*Craniophora* Snellen, 1867 is an Old World genus of the Acronictinae restricted to the Palaearctic, Oriental, Australian and Ethiopian regions (Fibiger et al. 2009). Most of the 20 described species occur in eastern Asia (Poole 1989; Han and Kononenko 2010). Only a few taxonomic studies of the genus have been undertaken (e.g., Hampson 1909; Kozhanchikov 1950; Chen 1999; Han and Kononenko 2010, Kononenko 2010), and most of the publications mentioning *Craniophora* are faunistic works or check lists (e.g., Draudt 1937, 1950; Inoue et al. 1982; Holloway 1989; Kononenko et al. 1998; Yoshimoto 1994; Kononenko 2005). A diagnosis of the genus is given by Fibiger et al. (2009) and Han and Kononenko (2010).

In the subfamily Acronictinae there are two main branches according to the external and genital features. The first branch consists of the genera *Acronicta* Ochsenheimer, *Gerbathodes* Warren, *Moma* Hübner, *Oxicesta* Hübner, *Simyra* Ochsenheimer, *Subleuconycta* Kozhanchikov with diverse external features, but with similar genitalia with a heavily-sclerotised clasping apparatus and a simple structure of the vesica. The second group consists of the genus *Craniophora* with very similar external features, weakly sclerotised valvae and more complex vesica than in the first group.

The majority of the taxa of *Craniophora* and *Acronicta* are separable without checking their genital structures. In smaller species groups, however, the separation of species using external features requires thorough study due to the similarity in forewing pattern and shared features of the typical noctuid maculation. Forewing traits shared by many *Craniophora* and *Acronicta* include the variably strong basal dash, and the black streak of the tornus situated in the submedial fold, often extending from the medial area or the postmedial line to the terminal line. Moreover, in the three main species-groups of *Craniophora*, such as the *pontica*-, *harmandi*- and the *fasciata*-groups (although these have not previously been proposed formally, the common external and genital features suggest it), identification of some species requires detailed study of the genitalia.

Additionally, the species of *Cranionycta* de Lattin, which is a distinct lineage, probably with intermediate position between *Acronicta* and *Craniophora* (distributed in the Russian Far East, China, Nepal) externally can also be very similar to *Craniophora*, although they are usually smaller with narrower wings than most of the *Craniophora* species. The main differences are found in the specific features of the genitalia of both sexes (see de Lattin 1949; Inoue and Sugi 1958; Kononenko et al. 1998); in the males of *Craniophora*, the most conspicuous features are the much broader, but shorter, less sclerotised valvae, slighter corona, more diverticulated or twisted and more or less larger vesica; in the females the corpus bursae is more developed, without signa.

## Method

Specimens were collected in China using ultraviolet light traps. Genital slides were prepared following standard techniques (abdominal integument cut lengthwise after KOH maceration male and female genitalia were dissected and mounted in euparal or in Canada balsam on glass slides). Additional material for comparison was borrowed from the Hungarian Natural History Museum, Budapest (HNHM). Additionally, numerous genitalia dissections were examined, both material on loan and own material.

The genital slides were digitalized with an Olympus SZX12 zoom stereo microscope with an Olympus DP 70 digital microscope camera in the Hungarian Natural History Museum, Budapest. After the digitalization, the pictures were converted to greyscale mode and the unnecessary grey background was deleted by photo editing software (Gimp). The habitus pictures were taken with Nikon D200 with Micro-Nikkor 200mm F/4 lens and Nikon D90 with Nikkor 200mm F/4 lens, after deleted the background by software.

The authors of all of the newly described taxa are the authors of this paper; however, *Craniophora sichuanensis* sp. n. is described in co-authorship with Aidas Saldaitis.

## Systematic part

### Genus *Craniophora* Snellen, 1867

*Craniophora* Snellen, 1867, *De Vlinders van Nederland, Macrolepidoptera systematisch beschreven*: 262.

**Type species.** *Noctua ligustri* [Denis et Schiffermüller], 1775, *Ankündung eines systematischen Werkes von den Schmetterlingen der Wienergegend*: 70.

### Craniophora fujianensis

Kiss & Gyulai sp. n.

http://zoobank.org/E032E47F-3F27-44A3-8AEA-78C7683AC46B

http://species-id.net/wiki/Craniophora_fujianensis

Figs 1 , 7 , 8

#### Type material.

**Figures 1–6.**
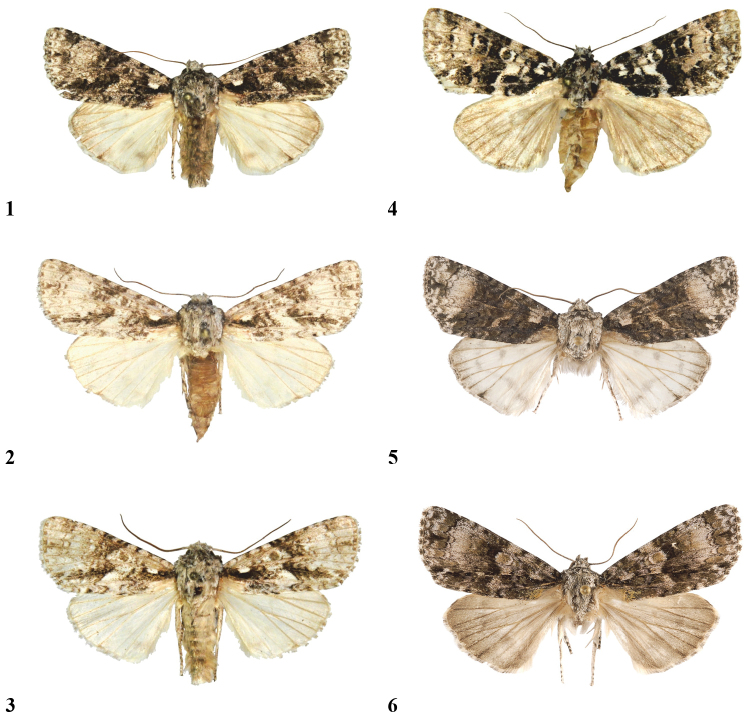
Adults. **1** *Craniophora fujianensis* sp. n., holotype, male, China, Fujian, coll. P. Gyulai **2** *Craniophora fujianensis hainanensis* ssp. n., holotype, male, China, Hainan, coll. P. Gyulai **3** *Craniophora fujianensis hainanensis* ssp. n., paratype, male, China, Hainan, coll. G. Ronkay **4** *Craniophora sichuanensis* sp. n., holotype, male, China, Sichuan, coll. P. Gyulai **5** *Craniophora harmandi*, male, Nepal, coll. HNHM **6** *Craniophora praeclara*, male, North Korea, coll. HNHM.

**Holotype:** Male (Fig. 1), China, Fujian, Dai Mao Shan, 20 km NW of Longyan, 25°32'N, 116°51'E, 1300 m, 21–30.Nov.2004, leg V. Siniaev and team; slide No.: 3207 Gyulai (coll. P. Gyulai, to be deposited in Hungarian Natural History Museum (HNHM), Budapest). **Paratypes:** None. We exclude specimens of *Craniophora fujianensis* from Hainan, China, as these represent a separate subspecies, described below.

#### Diagnosis and description.

**Figures 7–12.**
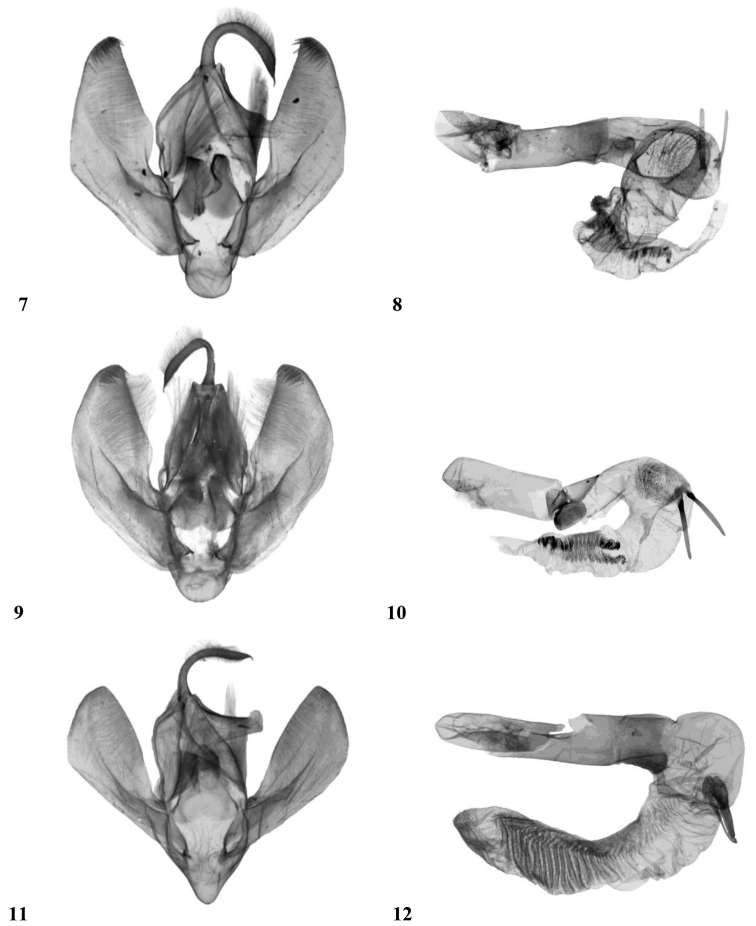
Male genitalia. **7, 8** *Craniophora fujianensis* sp. n., holotype, male genitalia, China, Fujian, slide No.: 3207 Gyulai, coll. P. Gyulai **7** valvae **8** aedeagus. **9, 10** *Craniophora fujianensis hainanensis* ssp. n., holotype, male, China, Hainan, slide No.: 3502 Gyulai, coll. P. Gyulai **9** valvae **10** aedeagus **11, 12** *Craniophora sichuanensis* sp. n., holotype, male genitalia, China, Sichuan, slide No.: 2883 Gyulai, coll P. Gyulai **11** valvae **12** aedeagus.

**Figures 13–16.**
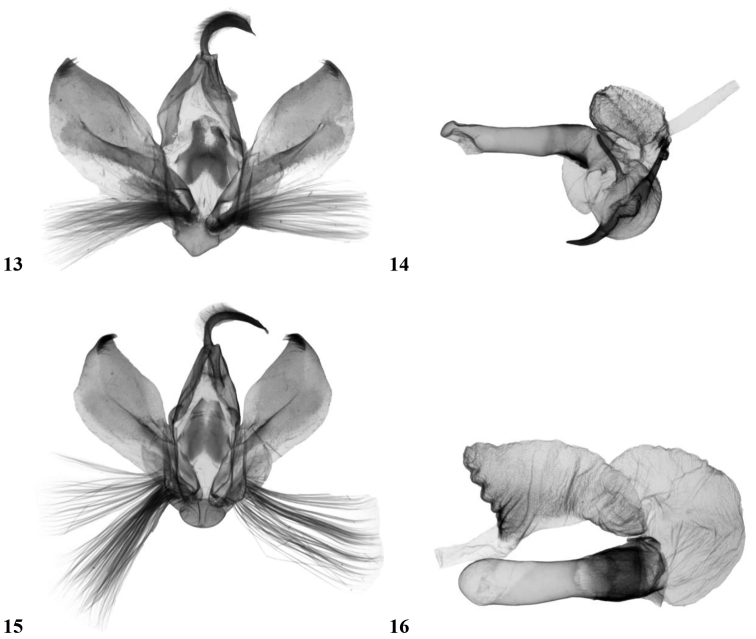
Male genitalia. **13, 14** *Craniophora harmandi*, male genitalia, Nepal, slide No.: KA138m, coll. HNHM **13** valvae **14** aedeagus. **15, 16** *Craniophora praeclara*, male genitalia, North Korea, slide No.: KA054m, coll. HNHM **15** valvae **16** aedeagus.

Wingspan 37 mm. *Craniophora fujianensis* resembles *Craniophora praeclara* (Graeser, 1890) (Fig. 6) and especially *Craniophora harmandi* (Poujade, 1898) (Fig. 5) externally. Reliable separation of the three taxa does not require genitalic study, since *Craniophora fujianensis* exhibits unique external characteristics. The shared features of the two related taxa are the more or less similar forewing pattern and noctuid maculation, the presence of the strong or weaker black streaks in the basal area, in the termen and the tornus (the latter streak in the submedial fold, regularly from the medial area or the postmedial line towards the terminal line; the oblique, wavy antemedial line, the double, crenulate postmedial line and the less wavy whitish-grey subterminal line. *Craniophora fujianensis* can be distinguished from *Craniophora praeclara* and *Craniophora harmandi* by its more uniform vestiture of thorax, light brownish-grey (and not chequered white) forewing fringe and the less evenly broad, somewhat shorter blackish streak extending through the submedial fold from the medial area outwards to the lowest part of the terminal line. In comparison with *Craniophora harmandi*, the new species has a more unicolorous, lighter brownish-grey forewing ground colour; lighter, more obsolescent, narrower dark suffusion in the medial area, conspicuous clear white colouration of the small quadrangular basal spot, which is not confluent with the whitish spot of the costal field; rather ashy grey (and not white), less conspicuous comma-like tiny spot beside the claviform stigma; the stigmata are smaller, the orbicular spot is not evenly white encircled. *Craniophora fujianensis* is distinguished from *Craniophora praeclara* by its smaller average size, more unicolorous, lighter brownish-grey forewing ground colour, without mossy green shades; lighter, more obsolescent, narrower dark suffusion in the medial area; clear white small quadrangular basal spot; less crenulate postmedial line, much smaller, blackish-filled stigmata and especially by the almost white hindwing of the male. **Male genitalia** (Figs 7, 8): a close relationship with *Craniophora harmandi* (Figs 13, 14) is evident; however, the differences are very conspicuous. *Craniophora fujianensis* can be easily distinguished from both of the allied taxa by its much larger, longer uncus, larger juxta and vinculum, large bundle of long hairs on the tegumen, strikingly elongate, curved valvae with straighter dorsal and almost evenly curved (with one angle medially) ventral costa and broader corona with much longer setae. The aedeagus is larger, the vesica ventrally curved; the two medial spines are straight, almost evenly thin and parallel, the third, weaker medial spine weakly sclerotised and hardly visible; whereas the two large spines are oppositely positioned in *Craniophora harmandi*, and the third, medial, cornutus is weaker than the others but stronger than in *Craniophora fujianensis*; *Craniophora praeclara* has no cornuti in the vesica (Figs 15, 16). Additionally, *Craniophora fujianensis* has a tiny semiglobular, sclerotised medial diverticulum, finely serrate on its surface, from which a longitudinal, wavy-ribbed, sclerotised area is situated towards the terminal section of the vesica.

**Female.** Unknown.

#### Etymology.

The species name refers to Fujian Province, China, where the species was discovered.

#### Distribution.

The species is known from Fujian and Hainan Provinces, China, with the nominate subspecies known only from the type locality in Fujian; subspecies *hainanensis* occurs in Hainan. *Craniophora fujianensis* is the allopatric sister taxa of *Craniophora harmandi*, which occurs from the western Himalaya to Taiwan, in the region with monsoonic influence.

### Craniophora fujianensis hainanensis

Kiss & Gyulai ssp. n.

http://zoobank.org/3A6BE9D1-5170-4B00-925A-5A8E250C5909

http://species-id.net/wiki/Craniophora_fujianensis_hainanensis

Figs 2 , 3 , 9 , 10

#### Type material.

**Holotype:** Male (Fig. 2), China, prov. Hainan, Wuzhi Shan, 1333 m, 03–10.Jan.2008, leg local collector; slide No.: 3502 Gyulai (coll. P. Gyulai, to be deposited in HNHM, Budapest). **Paratype:** Male (Fig. 3), same data as holotype; slide No.: 3209 Gyulai (coll. G. Ronkay, Budapest, Hungary).

#### Diagnosis and description.

*Craniophora fujianensis hainanensis* is endemic to the island of Hainan. It can be separated at first sight from similar *Craniophora* by its whitish-ochreous forewing ground colour, indistinct wing pattern, with a more double-angled inner edge of the medial fascia, the whitish fringe and the conspicuous clear white hindwing. Wingspan 35–38 mm. **Male genitalia** (Figs 9, 10): In the male genitalia, ssp. *hainanensis* has a somewhat shorter uncus, with the ventral costa evenly rounded, and a distally more dilated valvae compared to the nominate subspecies. More specimens are needed to evaluate if these differences represent individual or subspecific variation. The two thin medial spines of the vesica are not parallel, but V-shaped, arising from the same sclerotised plate; the tiny semiglobular, medial diverticulum is hardly visible because the surface is not sclerotised or spinulose; the longitudinal, wavy-ribbed, sclerotised area towards the end of the vesica is bifurcate anteriorly then confluent.

**Female.** Unknown.

#### Etymology.

The name refers to the island of Hainan where this taxon occurs.

#### Distribution.

The subspecies is known only from the type-locality, China, Hainan Island.

### Craniophora sichuanensis

Kiss, Gyulai & Saldaitis sp. n.

http://zoobank.org/E24039A7-3A32-448D-BEE0-C56FED2881A4

http://species-id.net/wiki/Craniophora_sichuanensis

Figs 4 , 11 , 12

#### Type material.

**Holotype:** Male (Fig. 4), China, W. Sichuan, road Yaan/Kangding, Erlang Shan Mt., 2200 m, 02.Aug.2011, 29°87.340"N, 102°30.970"E, leg. Floriani and Saldaitis; slide No.: 2883 Gyulai (coll. P. Gyulai, to be deposited in HNHM, Budapest). **Paratypes:** None.

#### Diagnosis and description.

Wingspan 32 mm. Externally most similar to *Craniophora harmandi* and to a lesser degree to *Craniophora fujianensis*. The shared features with the two related taxa are the more or less similar forewing pattern and noctuid maculation and the less sinuous whitish-grey subterminal line. It can be distinguished from *Craniophora fujianensis* by its smaller size, with a wingspan of 32 mm compared to 35–38 mmin the two subspecies of *Craniophora fujianensis* and 33–40 mm in *Craniophora harmandi*; the slight black circle in the centre of the thoracic tuft; the white, curved, fine, comma-like basal mark (which is not quadrangular as in the two related taxa); the conspicuous, clear white inner stripe of the medial area along the broad black medial fascia; the more recognisable white outline of the orbicular and reniform stigmata; the longer basal black streak, the diluted blackish streak extending in the submedial fold from the middle of the medial line outward to the lowest part of the terminal line (tornal area) and the more uniform, light brownish-grey hindwing with a faint dark-brown discal spot, sinuous medial line and darker suffused terminal area. Additionally, in comparison with *Craniophora harmandi*, *Craniophora sichuanensis* has darker and narrower dark suffusion in the medial area, and lacks the large whitish area extending outward from the reniform stigma toward the apex and in the postmedial line. *Craniophora sichuanensis* is more distinct from *Craniophora fujianensis*, especially in the conspicuous clear white inner third of medial area. **Male genitalia** (Figs 11, 12): Uncus almost evenly slender and apically hooked, valvae spatulate, lacking corona, vesica almost even in width with two equally long, weak, slender spines and one shorter, broader, stout cornutus and the broad, sclerotised distal area covered by numerous almost straight parallel ribs. These genitalia features, as well as the overall smaller male genitalia, shorter, more asymmetrical, medially broadened valvae, and V-shaped vinculum, separate the new species from the two close relatives.

**Female.** Unknown.

#### Etymology.

The species name refers to the type locality in the Province of Sichuan, China.

#### Distribution.

The new species is known only from the Erlang Shan at the eastern edge of the Tibetan plateau in China’s Sichuan province. The single male was collected at ultraviolet light. The new species appeared with a very local distribution, as it was discovered in only one valley in mountainous region. The new species was collected in virgin mixed forest habitat dominated by various broad-leaved trees such as oaks (*Quercus dentata* Thunb., *Quercus glauca* Thunb.), poplars (*Populus cathayana* Rheder, *Populus simonii* Carrière), elm (*Ulmus parvifolia* Jacq.), rhododendrons (*Rhododendron brachycarpum* D. Don ex G. Don, *Rhododendron dauricum* L.), and bamboos (*Phyllostachys* ssp., *Borinda* ssp., *Fargesia* spp.). Adults are on the wing with many other late summer Noctuidae species, such as *Pareuplexia chalybeate* (Moore, 1867), *Blepharosis bryocharis* Boursin, 1964, *Blepharosis lamida* (Draudt, 1950) and *Amphipyra amentet* Babics, Benedek & Saldaitis, 2013.

## Supplementary Material

XML Treatment for Craniophora fujianensis

XML Treatment for Craniophora fujianensis hainanensis

XML Treatment for Craniophora sichuanensis
